# Global Prevalence and Potential Influencing Factors of COVID-19 Vaccination Hesitancy: A Meta-Analysis

**DOI:** 10.3390/vaccines10081356

**Published:** 2022-08-19

**Authors:** Jonny Karunia Fajar, Malik Sallam, Gatot Soegiarto, Yani Jane Sugiri, Muhammad Anshory, Laksmi Wulandari, Stephanie Astrid Puspitasari Kosasih, Muhammad Ilmawan, Kusnaeni Kusnaeni, Muhammad Fikri, Frilianty Putri, Baitul Hamdi, Izza Dinalhaque Pranatasari, Lily Aina, Lailatul Maghfiroh, Fernanda Septi Ikhriandanti, Wa Ode Endiaverni, Krisna Wahyu Nugraha, Ory Wiranudirja, Sally Edinov, Ujang Hamdani, Lathifatul Rosyidah, Hanny Lubaba, Rinto Ariwibowo, Riska Andistyani, Ria Fitriani, Miftahul Hasanah, Fardha Ad Durrun Nafis, Fredo Tamara, Fitri Olga Latamu, Hendrix Indra Kusuma, Ali A. Rabaan, Saad Alhumaid, Abbas Al Mutair, Mohammed Garout, Muhammad A. Halwani, Mubarak Alfaresi, Reyouf Al Azmi, Nada A. Alasiri, Abeer N. Alshukairi, Kuldeep Dhama, Harapan Harapan

**Affiliations:** 1 Brawijaya Internal Medicine Research Center, Department of Internal Medicine, Faculty of Medicine, Universitas Brawijaya, Malang 65145, Indonesia; 2 Department of Pathology, Microbiology and Forensic Medicine, School of Medicine, The University of Jordan, Amman 11942, Jordan; 3 Department of Clinical Laboratories and Forensic Medicine, Jordan University Hospital, Amman 11942, Jordan; 4 Department of Translational Medicine, Faculty of Medicine, Lund University, 22184 Malmö, Sweden; 5 Division of Allergy and Immunology, Department of Internal Medicine, Faculty of Medicine, Universitas Airlangga, Surabaya 60286, Indonesia; 6 Department of Pulmonology and Respiratory Medicine, Faculty of Medicine, Universitas Brawijaya, Malang 65145, Indonesia; 7 Division of Allergy and Immunology, Department of Internal Medicine, Faculty of Medicine, Universitas Brawijaya, Malang 65145, Indonesia; 8 Department of Pulmonology and Respiratory Medicine, Faculty of Medicine, Universitas Airlangga, Surabaya 60286, Indonesia; 9 Faculty of Medicine, Universitas Brawijaya, Malang 65145, Indonesia; 10 Department of Urology, Faculty of Medicine, Universitas Brawijaya, Malang 65145, Indonesia; 11 Faculty of Matematics and Sciences, Institut Pertanian Bogor, Bogor 16680, Indonesia; 12 School of Veterinary Medicine and Biomedicine, Institut Pertanian Bogor, Bogor 16680, Indonesia; 13 Faculty of Economy and Business, Universitas Airlangga, Surabaya 60286, Indonesia; 14 Faculty of Public Health, Universitas Airlangga, Surabaya 60286, Indonesia; 15 Faculty of Pharmacy, Universitas Airlangga, Surabaya 60286, Indonesia; 16 Faculty of Veterinary Medicine, Universitas Airlangga, Surabaya 60286, Indonesia; 17 Faculty of Medicine, Universitas Airlangga, Surabaya 60286, Indonesia; 18 Faculty of Economy and Business, Universitas Brawijaya, Malang 65145, Indonesia; 19 Faculty of Economics and Business, Riau University, Pekanbaru 28293, Indonesia; 20 Faculty of Administrative Science, Universitas Brawijaya, Malang 65145, Indonesia; 21 Faculty of Animal Science, Universitas Brawijaya, Malang 65145, Indonesia; 22 Medical Research Unit, School of Medicine, Universitas Syiah Kuala, Banda Aceh 23111, Indonesia; 23 Faculty of Mathematics and Natural Sciences, Universitas Syiah Kuala, Darussalam, Banda Aceh 23111, Indonesia; 24 Faculty of Tarbiyah and Teacher Training, Universitas Islam Negeri Ar-Raniry, Banda Aceh 23111, Indonesia; 25 Molecular Diagnostic Laboratory, Johns Hopkins Aramco Healthcare, Dhahran 31311, Saudi Arabia; 26 College of Medicine, Alfaisal University, Riyadh 11533, Saudi Arabia; 27 Department of Public Health and Nutrition, The University of Haripur, Haripur 22610, Pakistan; 28 Administration of Pharmaceutical Care, Al-Ahsa Health Cluster, Ministry of Health, Al-Ahsa 31982, Saudi Arabia; 29 Research Center, Almoosa Specialist Hospital, Al Mubarrazs 36342, Saudi Arabia; 30 College of Nursing, Princess Norah Bint Abdulrahman University, Riyadh 11564, Saudi Arabia; 31 School of Nursing, Wollongong University, Wollongong, NSW 2522, Australia; 32 Nursing Department, Prince Sultan Military College of Health Sciences, Dhahran 33048, Saudi Arabia; 33 Department of Community Medicine and Health Care for Pilgrims, Faculty of Medicine, Umm Al-Qura University, Makkah 21955, Saudi Arabia; 34 Department of Medical Microbiology, Faculty of Medicine, Al Baha University, Al Baha 4781, Saudi Arabia; 35 Department of Pathology and Laboratory Medicine, Sheikh Khalifa General Hospital, Umm Al Quwain 499, United Arab Emirates; 36 Department of Pathology, College of Medicine, Mohammed Bin Rashid University of Medicine and Health Sciences, Dubai 505055, United Arab Emirates; 37 Infection Prevention and Control, Eastern Health Cluster, Dammam 32253, Saudi Arabia; 38 Scientific Advisory Council, InsanCare Group for Scientific Studies and Specialized Business Solutions, Riyadh 13313, Saudi Arabia; 39 Department of Medicine, King Faisal Specialist Hospital and Research Center, Jeddah 12713, Saudi Arabia; 40 Division of Pathology, ICAR–Indian Veterinary Research Institute, Izatnagar, Bareilly 243122, India; 41 Tropical Disease Centre, School of Medicine, Universitas Syiah Kuala, Banda Aceh 23111, Indonesia; 42 Department of Microbiology, School of Medicine, Universitas Syiah Kuala, Banda Aceh 23111, Indonesia

**Keywords:** COVID-19, vaccination, hesitancy, acceptance, prevalence

## Abstract

Countries worldwide have deployed mass COVID-19 vaccination drives, but there are people who are hesitant to receive the vaccine. Studies assessing the factors associated with COVID-19 vaccination hesitancy are inconclusive. This study aimed to assess the global prevalence of COVID-19 vaccination hesitancy and determine the potential factors associated with such hesitancy. We performed an organized search for relevant articles in PubMed, Scopus, and Web of Science. Extraction of the required information was performed for each study. A single-arm meta-analysis was performed to determine the global prevalence of COVID-19 vaccination hesitancy; the potential factors related to vaccine hesitancy were analyzed using a Z-test. A total of 56 articles were included in our analysis. We found that the global prevalence of COVID-19 vaccination hesitancy was 25%. Being a woman, being a 50-year-old or younger, being single, being unemployed, living in a household with five or more individuals, having an educational attainment lower than an undergraduate degree, having a non-healthcare-related job and considering COVID-19 vaccines to be unsafe were associated with a higher risk of vaccination hesitancy. In contrast, living with children at home, maintaining physical distancing norms, having ever tested for COVID-19, and having a history of influenza vaccination in the past few years were associated with a lower risk of hesitancy to COVID-19 vaccination. Our study provides valuable information on COVID-19 vaccination hesitancy, and we recommend special interventions in the sub-populations with increased risk to reduce COVID-19 vaccine hesitancy.

## 1. Introduction

Coronavirus disease 2019 (COVID-19) vaccination has been progressing globally since the beginning of 2021. Several types of vaccines, including inactivated, vector-based, messenger ribonucleic acid (mRNA), and protein subunit vaccines, are being administered to recipients [1]. Since the vaccines became available, there have been expectations of the COVID-19 pandemic ending, considering that previous vaccination programs have been effective in managing several infectious diseases such as rubella, mumps, measles, and polio. These vaccination programs have been proven to improve global health and the economy [2,3]. However, the probability of failure of any vaccination program should be assessed. A study reported that the barriers to effective vaccination programs include inconvenient and limited clinic hours for immunization, inadequate access to healthcare, high vaccine administration fees, and vaccine hesitancy [4]. Of these factors, vaccine hesitancy is considered one of the most critical [5]. Individuals who are hesitant to be immunized have a tendency to spread incorrect information about vaccination, which may influence people close to them to reject vaccines as well [6].

Vaccine hesitancy is commonly observed in the case of new vaccines or vaccine candidates [7,8]. This phenomenon was reported in the case of malaria [9], dengue [10], and Ebola [11]. The factors contributing to vaccine hesitancy are complex and may include a lack of awareness regarding disease prevention and socioeconomic status [12,13]. This phenomenon poses a dilemma to vaccine coverage. Moreover, governments—as the highest regulatory authority of any nation—seemingly do not provide special interventions to reduce hesitancy toward vaccination programs. It is observed in the guidelines on COVID-19 vaccination, that the primary recommendation only focused on dose allocation, outreach, delivery, and monitoring; there was no information on how to reduce COVID-19 vaccination hesitancy [14].

Regarding COVID-19 vaccination, several studies have been conducted to assess the prevalence of COVID-19 vaccine hesitancy and its associated predictors [7,8,15]. However, the findings were inconclusive with variability regarding the correlation between COVID-19 vaccine acceptance and the following: sociodemographic factors, vaccine confidence and trust regarding vaccine safety, complacency towards the disease, conspiracy beliefs towards COVID-19 vaccination and willingness to pay for the vaccine [7,16,17,18,19,20,21]. Therefore, a meta-analysis is necessary to determine the potential factors influencing COVID-19 vaccination hesitancy.

## 2. Materials and Methods

### 2.1. Study Design

During the period May–June 2022, we conducted a meta-analysis that followed the protocols of the Preferred Reporting Items for Systematic Review and Meta-Analysis (PRISMA) [22,23]. In line with the purpose of our study, we first performed an organized search of PubMed, Scopus, and Web of Science and, subsequently, collected the required information to calculate the global prevalence of vaccine hesitancy and effect estimates of the potential influencing factors. The PRISMA checklist for this review is provided in (Supplementary Materials). Additionally, data used in this review are available in Figshare (https://doi.org/10.6084/m9.figshare.20055539.v3, accessed on 6 December 2022) [23].

### 2.2. Eligibility Criteria

We determined the eligibility criteria before conducting the organized search. An article was included in the analysis if the following inclusion criteria were met: (1) whether it assessed the prevalence of COVID-19 vaccination hesitancy or (2) identified potential factors influencing COVID-19 vaccination hesitancy. Reviews, commentaries, letters to the editor, grey literature, and double publications were excluded.

### 2.3. Search Strategy and Data Extraction

As of 25 May 2022, we performed an organized search of PubMed, Scopus, and Web of Science. Prior to the search for the main outcomes, the potential factors associated with COVID-19 vaccination hesitancy were determined. We used keywords from the following medical subject headings: “vaccine”, “vaccination”, or “immunization”; “COVID-19” or “coronavirus disease 2019”; “hesitancy” or “acceptance”. We limited the organized search to the English language. If we found any duplication, we included the studies with larger sample sizes. Furthermore, we also conducted an organized search of the reference lists of the relevant articles to obtain additional papers. Thereupon, the following information was collected from the selected articles: (1) first author name, (2) year of publication, (3) study design, (4) study period, (5) Newcastle–Ottawa scale (NOS), (6) the prevalence of COVID-19 vaccination hesitancy, and (7) event rate of potential factors associated with COVID-19 vaccination hesitancy. Two independent teams, led by JKF and SAPK, conducted the article search and data extraction. Prior to the systematic search, the kappa statistic was used to measure the agreement between the two investigators. If the kappa statistic was greater than the *p*-value, agreement was established.

### 2.4. Assessment of the Methodological Quality

All potential articles for inclusion in the study were assessed for quality using NOS [24]. The quality was considered high, moderate, or low if the score was 7–9, 4–6, or 0–3, respectively. Low-quality articles were excluded from the analysis. Using a pilot form, the two independent teams, led by JKF and SAPK, conducted the NOS assessment, and any discrepancies were resolved through discussion.

### 2.5. Outcome Measures

The major outcomes were global prevalence and potential influencing factors of COVID-19 vaccination hesitancy. To identify the potential factors associated with vaccine hesitancy, we performed an initial organized search in PubMed, Scopus, and Web of Science. We identified the following potential factors: age group, gender, marital status, educational attainment, religion, employment status, healthcare-related job, socioeconomic status, urbanity, presence of children and elderly people at home, household size, and presence of family members with a medical background. Additionally, wearing masks, hand hygiene, compliance with physical distancing norms, smoking, history of chronic disease, personal history of COVID-19 diagnosis, COVID-19 diagnosis of a family member/friend, hospitalization due to COVID-19 among people in the same social circle, death owing to COVID-19 among people in the same social circle, safety conceptions about COVID-19 vaccines, and history of influenza vaccination in the past few years were also factors of interest.

### 2.6. Statistical Analysis

Before calculating the global prevalence of COVID-19 vaccination hesitancy and effect estimates of potential predictors of such hesitancy, we conducted an analysis of potential publication bias and heterogeneity among the studies. We analyzed the risk of publication bias using the Egger’s test, with a *p*-value of <0.05 suggesting the existence of publication bias. Furthermore, we performed an analysis of heterogeneity among studies using the Q test, with a *p*-value of <0.10 indicating heterogeneity; thus, a random effects model was applied for data analysis—in cases where there was no heterogeneity, a fixed-effects model was used. A single-arm meta-analysis was performed using the dichotomous covariate method to calculate the event rate from each study to discern the global prevalence of COVID-19 vaccination hesitancy. The effect estimate was presented as the event rate. The analysis was performed using the R package (RStudio version 4.1.1, R Studio, Boston, MA, USA). The effect estimates of potential factors associated with COVID-19 vaccination hesitancy were outlined in a forest plot as a pooled odds ratio and 95% confidence interval (OR, 95% CI).

## 3. Results

### 3.1. Selection of Studies

**Figure 1:**
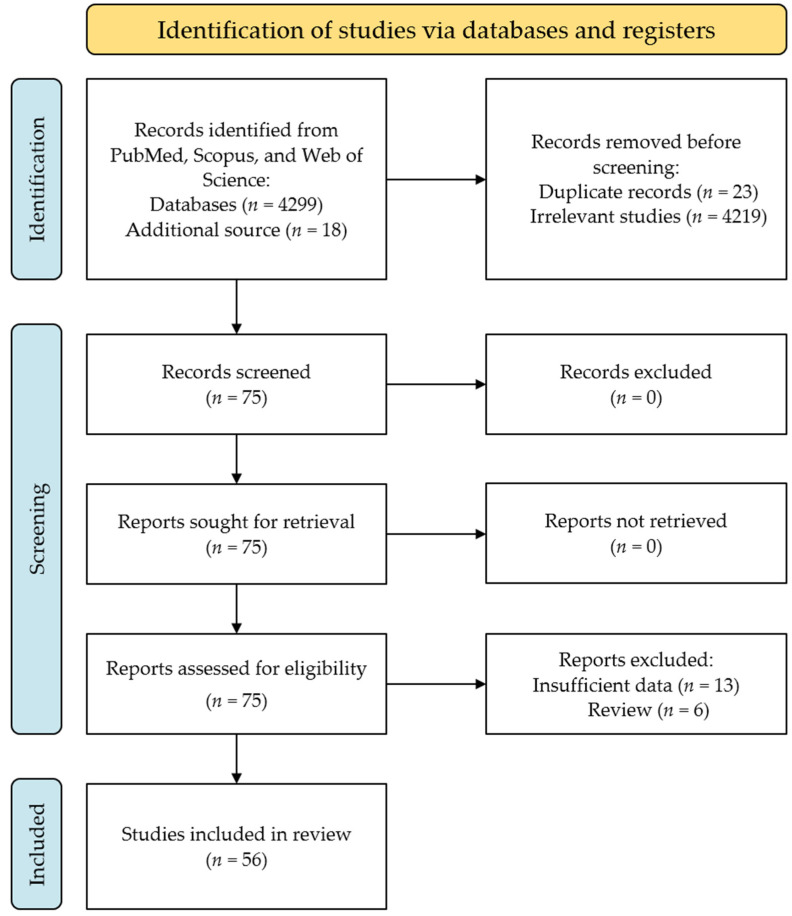
A flowchart of article selection in this review.

We retrieved 4299 potential papers from the databases mentioned and 18 from the reference lists of related articles. Of these, 23 papers were excluded owing to duplication and 4219 papers with irrelevant subjects. Thus, 75 articles were included in the full-text review. Subsequently, six reviews and thirteen articles were excluded because of insufficient data. Eventually, a total of 56 articles were included in the final analysis to calculate the global prevalence and potential influencing factors in COVID-19 vaccination hesitancy [19,25,26,27,28,29,30,31,32,33,34,35,36,37,38,39,40,41,42,43,44,45,46,47,48,49,50,51,52,53,54,55,56,57,58,59,60,61,62,63,64,65,66,67,68,69,70,71,72,73,74,75,76,77,78,79]. The plotting of article selection in our study is presented in (Figure 1), and the characteristics of the included articles are listed in (Table 1).

### 3.2. Global Prevalence of COVID-19 Vaccination Hesitancy

**Figure 2:**
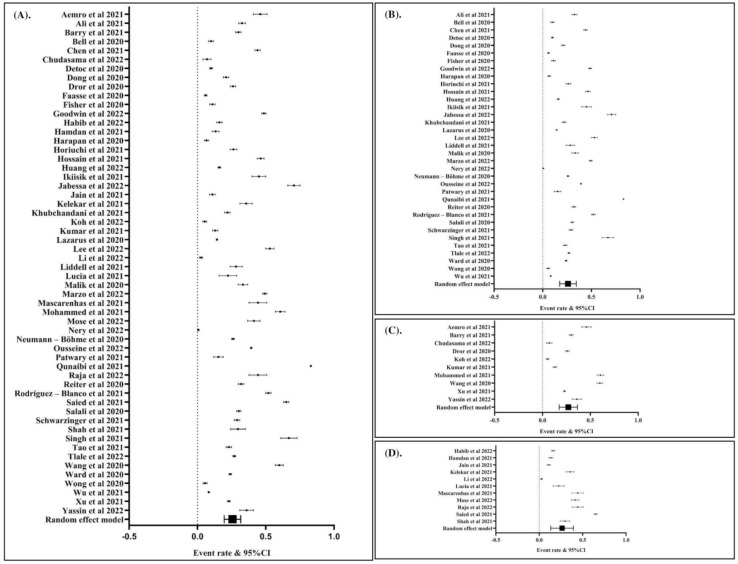
The global prevalence of hesitancy to COVID-19 vaccination (event rate: 0.25; 95% CI: 0.20, 0.32; *p* Egger: 1.2580; *p* heterogeneity: <0.0001; *p*: <0.0001) (**A**). The prevalence of hesitancy to COVID-19 vaccination among general population (event rate: 0.25; 95% CI: 0.18, 0.34; *p* Egger: 1.3090; *p* heterogeneity: <0.0001; *p*: <0.0001) (**B**). The prevalence of hesitancy to COVID-19 vaccination among healthcare workers (event rate: 0.26; 95% CI: 0.18, 0.37; *p* Egger: 0.7670; *p* heterogeneity: <0.0001; *p*: <0.0001) (**C**). The prevalence of hesitancy to COVID-19 vaccination among students (event rate: 0.25; 95% CI: 0.14, 0.40; *p* Egger: 1.2090; *p* heterogeneity: <0.0001; *p*: 0.0030) (**D**). The studies included are provided in the reference list [19,25,26,27,28,29,30,31,32,33,34,35,36,37,38,39,40,41,42,43,44,45,46,47,48,49,50,51,52,53,54,55,56,57,58,59,60,61,62,63,64,65,66,67,68,69,70,71,72,73,74,75,76,77,78,79].

To calculate the global prevalence of COVID-19 vaccination hesitancy, we included a total of 56 articles. Data analysis using the random effects model revealed that the global prevalence was 25% (event rate: 0.25; 95% CI: 0.19, 0.32; *p* Egger’s: 1.2710; *p* heterogeneity: <0.0001; *p* < 0.0001). The global prevalence of COVID-19 vaccination hesitancy is presented in (Figure 2A). In sub-group analysis, we found that the prevalence of COVID-19 vaccination hesitancy in the general population (Figure 2B), healthcare workers (Figure 2C), and students (Figure 2D) was 25%, 26%, and 25%, respectively.

### 3.3. Potential Factors Associated with COVID-19 Vaccination Hesitancy

**Figure 3:**
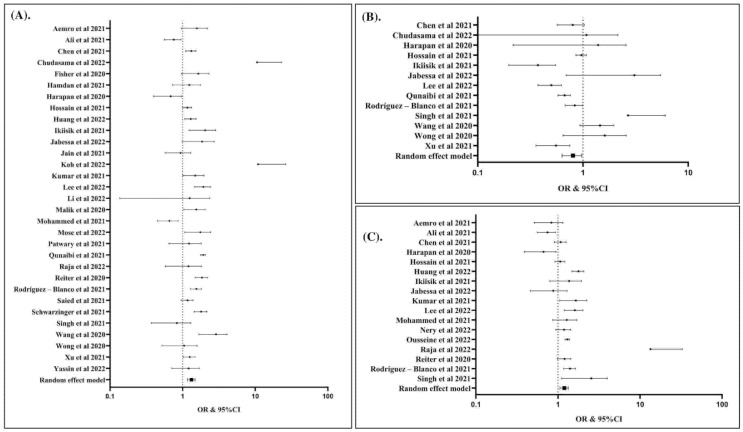
Female was associated with increased risk of hesitancy to COVID-19 vaccination compared to male (OR: 1.32; 95% CI: 1.17, 1.49; *p* Egger: 0.2840; *p* heterogeneity: <0.0001; *p*: <0.0001) (**A**). Individuals with age > 50 years was associated with lower risk of hesitancy to COVID-19 vaccination compared to individuals with age ≤ 50 years (OR: 0.79; 95% CI: 0.64, 0.98; *p* Egger: 0.2980; *p* Het: <0.0001; *p*: 0.0290) (**B**). Single individuals had higher risk of hesitancy to COVID-19 vaccination than married individuals (OR: 1.19; 95% CI: 1.06, 1.34; *p* Egger: 0.1950; *p* heterogeneity: <0.0001; *p*: 0.0040) (**C**). The studies included are provided in the reference list [19,25,26,27,28,29,30,31,32,33,34,35,36,37,38,39,40,41,42,43,44,45,46,47,48,49,50,51,52,53,54,55,56,57,58,59,60,61,62,63,64,65,66,67,68,69,70,71,72,73,74,75,76,77,78,79].

**Figure 4:**
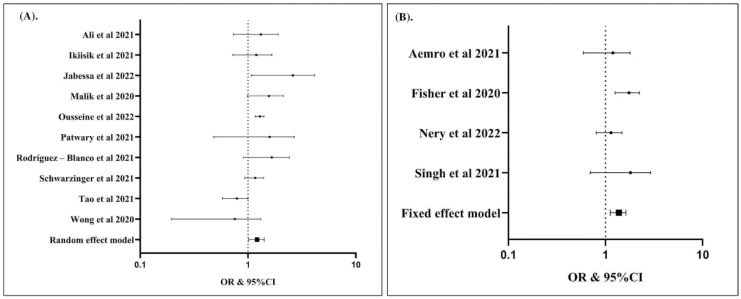
Unemployed individuals were associated with increased risk of hesitancy to COVID-19 vaccination compared to working individuals (OR: 1.20; 95% CI: 1.02, 1.42; *p* Egger: 0.1790; *p* heterogeneity: 0.0090; *p*: 0.0300) (**A**); individuals with household number ≥ 5 individuals had higher risk of hesitancy to COVID-19 vaccination (OR: 1.36; 95% CI: 1.13, 1.63; *p* Egger: 0.1680; *p* heterogeneity: 0.1620; *p*: 0.0010) (**B**). The studies included are provided in the reference list [19,25,26,27,28,29,30,31,32,33,34,35,36,37,38,39,40,41,42,43,44,45,46,47,48,49,50,51,52,53,54,55,56,57,58,59,60,61,62,63,64,65,66,67,68,69,70,71,72,73,74,75,76,77,78,79].

**Figure 5:**
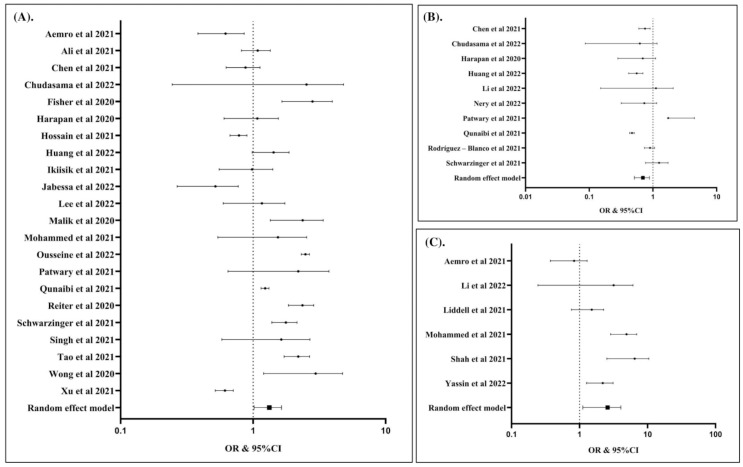
Individuals with the education levels < BSC had higher risk of hesitancy to COVID-19 vaccination than ≥ BSC (OR: 1.30; 95% CI: 1.03, 1.65; *p* Egger: 0.5260; *p* heterogeneity: <0.0001; *p*: 0.0300) (**A**); individuals having the healthcare-related job had lower risk of hesitancy to COVID-19 vaccination (OR: 0.68; 95% CI: 0.52, 0.89; *p* Egger: 0.3340; *p* heterogeneity: <0.0001; *p*: 0.0040) (**B**); Individuals considering that COVID-19 vaccines are not safe had higher risk of hesitancy to COVID-19 vaccination (OR: 2.24; 95% CI: 1.21, 4.14; *p* Egger: 0.7000; *p* heterogeneity: <0.0001; *p*: 0.0100) (**C**). The studies included are provided in the reference list [19,25,26,27,28,29,30,31,32,33,34,35,36,37,38,39,40,41,42,43,44,45,46,47,48,49,50,51,52,53,54,55,56,57,58,59,60,61,62,63,64,65,66,67,68,69,70,71,72,73,74,75,76,77,78,79].

**Figure 6:**
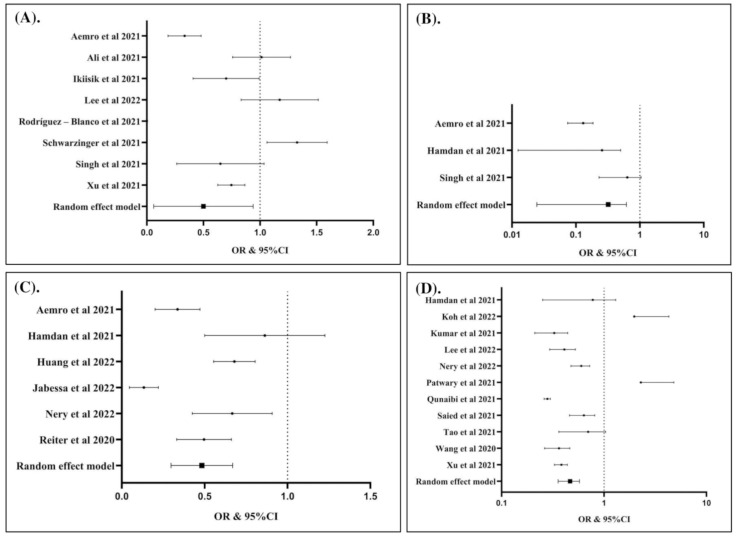
Individuals living with children in the home had lower risk of hesitancy to COVID-19 vaccination (OR: 0.37; 95% CI: 0.14, 0.99; *p* Egger: 1.4040; *p* heterogeneity: <0.0001; *p*: 0.0490) (**A**); individuals keeping physical distancing had lower risk of hesitancy to COVID-19 vaccination (OR: 0.23; 95% CI: 0.08, 0.65; *p* Egger: 0.8370; *p* heterogeneity: 0.0010; *p*: 0.0050 (**B**); individuals with history of COVID-19 test were associated with lower risk of hesitancy to COVID-19 vaccination (OR: 0.46; 95% CI: 0.31, 0.68; *p* Egger: 0.4340; *p* heterogeneity: <0.0001; *p*: <0.0001) (**C**); individuals with history of influenza vaccination in the past few years had lower risk of hesitancy to COVID-19 vaccination (OR: 0.46; 95% CI: 0.36, 0.58; *p* Egger: 0.3460; *p* heterogeneity: <0.0001; *p*: <0.0001) (**D**). The studies included are provided in the reference list [19,25,26,27,28,29,30,31,32,33,34,35,36,37,38,39,40,41,42,43,44,45,46,47,48,49,50,51,52,53,54,55,56,57,58,59,60,61,62,63,64,65,66,67,68,69,70,71,72,73,74,75,76,77,78,79].

The potential factors associated with COVID-19 vaccination hesitancy are summarized in (Table 2) and presented in (Figure 3, Figure 4, Figure 5 and Figure 6). Our analysis revealed that out of 25 factors, 12 of them were associated with COVID-19 vaccine hesitancy. The following factors were associated with a higher risk of vaccination hesitancy: being a woman (compared to man) (Figure 3A), being ≤50 years old (compared to those older than 50 years (Figure 3B), being single (compared to married individuals) (Figure 3C), being unemployed (compared to employed) (Figure 4A), living in a household with five or more individuals (compared to living in smaller households) (Figure 4B), having an educational attainment lower than an undergraduate degree (compared to those with an undergraduate degree or higher) (Figure 5A), having a non-healthcare-related job (compared to having a healthcare-related job) (Figure 5B), and considering COVID-19 vaccines to be unsafe (compared to those who consider COVID-19 vaccines to be safe) (Figure 5C).

In contrast, living with children at home (compared to having no child at home) (Figure 6A), maintaining physical distancing norms (compared to not following such norms) (Figure 6B), having ever tested for COVID-19 (compared to having never tested for COVID-19) (Figure 6C), and having a history of influenza vaccination in the past few years (compared to not having been vaccinated for influenza in the past few years) (Figure 6D) were associated with a lower risk of hesitancy to COVID-19 vaccination.

### 3.4. Source of Heterogeneity and Potential Publication Bias

Heterogeneity was not found for six variables (Christian religion, household size ≥ 5 individuals, family members with a medical background, smoking, hospitalization due to COVID-19 among people in the same social circle, and death owing to COVID-19 among people in the same social circle). Therefore, we used a fixed-effects model. Conversely, other variables were assessed using a random effects model. The Egger’s test was used to assess potential bias among the studies. Our pooled analyses revealed a risk of publication bias for the following covariates: the Christian religion, family members with a medical background, hospitalization due to COVID-19 among people in the same social circle, and death owing to COVID-19 among people in the same social circle (Table 2).

## 4. Discussion

Our study estimated the global prevalence of COVID-19 vaccination hesitancy at 25%. The current findings are consistent with those of previous meta-analyses, which estimated the prevalence of vaccine hesitancy in the general population at 26–42% [15,80,81,82,83]. In special populations, previous meta-analyses revealed that the hesitancy for COVID-19 vaccination was estimated at 24%, 27%, and 26% in multiple sclerosis patients, older people, and healthcare students, respectively [84,85,86]. Furthermore, hesitancy to receive a COVID-19 booster was reported at 21% in the general population [87]. Moreover, high rates of COVID-19 vaccine hesitancy were reported among the ethnic minorities in the UK [88]. Our estimate was in the range of extant literature. However, our study had a larger sample size, which might have provided a more accurate calculation. Moreover, in sub-group analysis, we also identified that the prevalence of COVID-19 vaccination hesitancy was 25%, 26%, and 25% in the general population, healthcare workers, and students, respectively. This study also identified the potential predictors of COVID-19 vaccine hesitancy, thereby providing more comprehensive evidence on this phenomenon.

The current study noted that the potential factors associated with COVID-19 vaccination hesitancy can be contextualized in terms of awareness, knowledge, and socioeconomic status. In the context of awareness of COVID-19 vaccination, we found that older people (>50 years), those living with children at home, individuals who have ever tested for COVID-19, and those with a history of influenza vaccination had a lower risk of COVID-19 vaccination hesitancy. In contrast, several factors, such as single marital status and unemployment, were associated with an increased risk of hesitancy toward COVID-19 vaccination.

The precise underlying factors contributing to COVID-19 vaccination hesitancy could not be defined clearly. However, some presumptions may explain these findings. Older individuals are more likely to suffer from one or more chronic diseases compared to younger people. In our previous investigation, we found that advanced age and comorbidity were associated with an increased risk of severity in COVID-19 patients [89]. Therefore, the possibility of an increased risk of severe COVID-19 might influence the awareness of such individuals and contribute to a lower risk of COVID-19 vaccination hesitancy in this group due to low levels of complacency [90].

Similarly, individuals living with children at home might be afraid of transmitting the virus to their children should they be infected with COVID-19. Therefore, it is reasonable that this group is less hesitant to receive COVID-19 vaccination. Interestingly, a similar impact was not observed in individuals living with elderly people at home. This ironic finding is supported by previous studies, which found that living with children was a crucial determinant of health-related behavior [91], whereas this was not the case for individuals living with elderly people [92].

Furthermore, individuals who have ever tested for COVID-19 and had a history of influenza vaccination might have had good practice in disease prevention. Disease screening and vaccination history have been shown to affect health behavior, which can possibly explain why this group is less averse to COVID-19 vaccination. Moreover, married individuals might engage in protective behavior toward their spouse; couples have mutual concern and might have a better life expectancy than single individuals. A previous study found that married individuals had better health behavior and a lower risk of mortality than single individuals [93]. Thus, single individuals might be more averse than married individuals to COVID-19 vaccination. This reason, in the context of poor health behavior, might also explain vaccine hesitancy in unemployed individuals.

Our study also found that individuals with lower educational levels and those who consider COVID-19 vaccines to be unsafe had a higher risk of COVID-19 vaccination hesitancy. In contrast, individuals with healthcare-related jobs had a lower risk of COVID-19 vaccination hesitancy. The association between higher educational levels, knowledge of disease prevention, and vaccine acceptance or hesitancy has been widely investigated [16,94,95,96]. Individuals with higher educational levels and healthcare-related jobs might have adequate information on the global pandemic and consider vaccination to be a great step toward ending the pandemic, which can explain why this group had a lower risk of vaccine hesitancy. Our current findings are supported by previous studies in the context of dengue, Ebola, and monkeypox vaccines. Those studies also showed that knowledge of disease prevention and good health practices had a significant impact on the acceptance of vaccine candidates [97,98,99,100,101].

Although we could not elucidate the role of socioeconomic status in COVID-19 vaccination hesitancy in this study, we found that some factors related to socioeconomic status, such as unemployment and household size (≥5 individuals), were associated with COVID-19 vaccination hesitancy. Socioeconomic status has been proven to affect health-related behavior [102]. Individuals with a low socioeconomic status might lack knowledge of the pandemic and the role of vaccination in the pandemic. Moreover, individuals with a low socioeconomic status might also lack social interaction; therefore, they might lack adequate knowledge concerning disease prevention, which could contribute to COVID-19 vaccination hesitancy. Previous meta-analyses in this context did not assess the role of socioeconomic status in COVID-19 vaccination hesitancy. However, in other settings, such as in the case of dengue vaccines, socioeconomic status was found to affect vaccination acceptance [100].

To the best of our knowledge, this meta-analysis is the first comprehensive study to assess COVID-19 vaccination hesitancy worldwide. In sub-group analysis, our study identified similar prevalence rates of hesitancy to COVID-19 vaccination in the general population, healthcare workers, and students; suggesting that interventions to reduce the risk of COVID-19 vaccination hesitancy in those populations do not need to be differentiated. In addition to reporting the global prevalence, we also identified the potential factors associated with hesitancy to receive COVID-19 vaccination. Although the COVID-19 vaccination program targets the global population, some people have been hesitant to receive the vaccine. Our study identified the factors associated with such hesitancy, thereby shedding light on the populations that require special attention in order for the vaccination program to be successful. We recommend customized interventions and education for these special populations. A study suggested that customized effective, ethical, and evidence-based communication may be able to increase the acceptance of the COVID-19 vaccination [103]. This customized intervention was suggested to provide by community leaders and healthcare practitioners to establish the trust of COVID-19 vaccination [88]. Moreover, a recent study also reported that providing the population with reliable information regarding the COVID-19 pandemic and the COVID-19 vaccination was associated with the increased rate of vaccination acceptance among the Israeli parents [104]. On the other hand, while we have provided the valuable information on the factors associated with the risk COVID-19 vaccine hesitancy, it should be realized that the main factor driving individuals to be able to receive the vaccinations is the proven effectiveness and safety of vaccines in well-documented long-term studies. However, among those exhibiting COVID-19 vaccination hesitancy, there are people who outright refuse vaccination. Therefore, further studies should be performed with a focus on this group.

There are some potential limitations of our study. First, a meta-analysis is a methodological approach conducted by calculating the crude effect of the related factors to determine the evidence. However, the impact of potential confounding factors is difficult to evaluate. In the current study, potential confounding factors such as the types of COVID-19 vaccine, government regulations, source of information regarding COVID-19 vaccination, and environmental factors were not included; therefore, our findings should be interpreted carefully. We reported that considering the COVID-19 vaccine to be unsafe was one of the factors associated with increased risk of COVID-19 vaccination hesitancy. Considering that the different types of COVID-19 vaccine have different side effects; the factors of the type of vaccine might also govern the final findings. Moreover, the government regulations in several countries have implemented COVID-19 vaccination as a condition of administration, and the regulations in each country may have differences; thereby, this circumstance may also affect the final findings of this study. Therefore, we reiterate the importance of the basic tenet in studying the phenomenon of vaccine hesitancy, which is the time, context, place, and type specificity. All these peculiarities need to be considered in the efforts aiming to fathom the determinants of vaccine hesitancy [105]. Second, our study involved a multi-national population, and the knowledge of disease prevention among people with similar socioeconomic status and educational level might vary in each region. Third, all the papers included in our study had an observational research design. Therefore, further studies including randomized controlled trials are required in order to obtain better levels of evidence.

## 5. Conclusions

Our study estimated the global prevalence of COVID-19 vaccination hesitancy at 25%. It also recommended special interventions to minimize COVID-19 vaccination hesitancy among unmarried individuals, women, people with low educational levels, the unemployed, people living in households with five or more individuals, and those who believe COVID-19 vaccines to be unsafe.

## Figures and Tables

**Table 1:** Baseline characteristics of articles included in our analysis [19,25,26,27,28,29,30,31,32,33,34,35,36,37,38,39,40,41,42,43,44,45,46,47,48,49,50,51,52,53,54,55,56,57,58,59,60,61,62,63,64,65,66,67,68,69,70,71,72,73,74,75,76,77,78,79].

Author and Year	Country	Sample Size	Study Period	Population	Funding	NOS ^1^
Aemro et al., 2021 [25]	Ethiopia	418	May–June 2021	Healthcare workers	No funding	5
Ali et al., 2021 [26]	Bangladesh	1134	January 2021	General population	No funding	6
Barry et al., 2021 [27]	Saudi Arabia	1512	November 2020	Healthcare workers	No funding	6
Bell et al., 2020 [28]	England	1252	April–May 2020	General population	London School of Hygiene and Tropical Medicine	6
Chen et al., 2021 [29]	China	2531	January 2021	General population	NA	7
Chudasama et al., 2022 [30]	Multinational	275	April–July 2021	Healthcare workers	NA	6
Detoc et al., 2020 [31]	France	3259	March–April 2020	General population	NA	5
Dong et al., 2020 [32]	China	1236	June–July 2020	General population	Chinese University of Hong Kong	6
Dror et al., 2020 [33]	Israel	1661	2020–2022	Healthcare workers	NA	5
Faasse et al., 2020 [34]	Australia	2232	March 2020	General population	UNSW Science Goldstar (2020)	5
Fisher et al., 2020 [35]	US	991	April 2020	General population	Agency for Healthcare Research and Quality	7
Goodwin et al., 2022 [36]	Multinational	3059	December 2020–January 2021	General population	Ariel University, JSPS KAKENSHI, Hungaria National Excellence Program	6
Habib et al., 2022 [37]	Saudi Arabia	1445	August–October 2021	Students	King Saud University	7
Bou Hamdan et al., 2021 [38]	Lebanon	758	May–June 2021	Students	No funding	7
Harapan et al., 2020 [39]	Indonesia	1359	March–April 2020	General population	No funding	6
Horiuchi et al., 2021 [40]	Japan	1200	May–June 2021	General population	No funding	7
Hossain et al., 2021 [41]	Bangladesh	1497	February 2021	General population	No funding	6
Huang et al., 2022 [42]	China	4227	January–March 2021	General population	National Health Commission of the People’s Republic of China	7
Ikiisik et al., 2021 [43]	Turkey	384	December 2020	General population	NA	7
Jabessa et al., 2022 [44]	Ethiopia	350	August–September 2021	General population	No funding	6
Jain et al., 2021 [45]	India	1068	February–March 2021	Students	No funding	6
Kelekar et al., 2021 [46]	US	408	September–December 2020	Students	NA	6
Khubchandani et al., 2021 [47]	US	1878	June 2020	General population	No funding	8
Koh et al., 2022 [48]	Singapore	528	May–June 2021	Healthcare workers	No funding	6
Kumar et al., 2021 [49]	Qatar	1414	October–November 2020	Healthcare workers	Qatar National Library	5
Lazarus et al., 2020 [50]	Multinational	13,426	June 2020	General population	City University of New York	6
Lee et al., 2022 [51]	South Korea	1016	January 2021	General population	No funding	6
Li et al., 2022 [52]	China	721	June 2021	Students	Xuzhou Medical University	7
Liddell et al., 2021 [53]	Australia	437	June 2021	General population	UNSW Sydney/Australian Red Cross	6
Lucia et al., 2021 [54]	US	167	NA	Students	No funding	5
Malik et al., 2020 [55]	US	672	May 2020	General population	Yale Institute for Global Health	8
Marzo et al., 2022 [56]	Multinational	5260	February–May 2021	General population	No funding	7
Mascarenhas et al., 2021 [57]	US	245	2020	Students	No funding	6
Mohammed et al., 2021 [58]	Ethiopia	614	March–July 2021	Healthcare workers	No funding	7
Mose et al., 2022 [59]	Ethiopia	420	March 2021	Students	No funding	6
Nery et al., 2022 [60]	Brazil	2537	November 2020–January 2021	General population	Brazilian Ministry of Health	8
Neumann-Böhme et al., 2020 [61]	Multinational	7664	April 2020	General population	European Union’s Horizon 2020 research and innovation programme	6
Ousseine et al., 2022 [62]	France	15,427	February–April 2021	General population	National Agency for Research on AIDS and Viral Hepatitis (ANRS)	6
Patwary et al., 2021 [19]	Bangladesh	543	July–August 2021	General population	No funding	6
Qunaibi et al., 2021 [63]	Multinational	36,220	January 2021	General population	No funding	7
Raja et al., 2022 [64]	Sudan	217	June–July 2021	Students	No funding	5
Reiter et al., 2020 [65]	US	2006	May 2020	General population	National Center for Advancing Translational Sciences	7
Rodríguez-Blanco et al., 2021 [66]	Spain	2494	November–December 2020	General population	No funding	6
Saied et al., 2021 [67]	Egypt	2133	January 2021	Students	NA	7
Salali et al., 2020 [68]	Multinational	5024	May 2020	General population	No funding	6
Schwarzinger et al., 2021 [69]	France	1942	July 2020	General population	French Public Health Agency	7
Shah et al., 2021 [70]	India	274	February 2021	Students	NA	7
Singh et al., 2021 [71]	Hong Kong	245	May 2021	General population	Tung Foundation	7
Tao et al., 2021 [72]	China	1392	November 2020	General population	National Key Research and Development Project of China	7
Tlale et al., 2022 [73]	Botswana	4952	February 2021	General population	No funding	6
Wang et al., 2020 [74]	Hong Kong	806	February–March 2020	Healthcare workers	No funding	6
Ward et al., 2020 [75]	France	5018	April 2020	General population	Agence Nationale de la Recheche and the CNRS	8
Wong et al., 2020 [76]	Malaysia	1159	April 2020	General population	Ministry of Education Malaysia	8
Wu et al., 2021 [77]	China	29,925	August 2021	General population	National Social Science Fund of China	7
Xu et al., 2021 [78]	China	5247	January 2021	Healthcare workers	Health Commission of Chongqing municipal, China	6
Yassin et al., 2022 [79]	Sudan	365	April 2021	Healthcare workers	NA	6

^1^ NOS: Newcastle–Ottawa scale; all selected studies were based on a cross-sectional design.

**Table 2:** The potential factors associated with the hesitancy to COVID-19 vaccination.

Covariates	Hesitancy/Total (n [%])	NS	*p* Egger	*p* Het	OR	95% CI	*p*
Age group (years)							
≤30	6568/14,356 [45.8]	16	0.2320	<0.0001	1.14	0.98–1.32	0.0870
31–40	5097/11,335 [45.0]	16	0.2360	<0.0001	1.09	0.94–1.26	0.2630
41–50	3034/6536 [46.4]	15	0.2730	<0.0001	0.88	0.74–1.06	0.1760
>50	2034/4677 [43.5]	13	0.2980	<0.0001	0.79	0.64–0.98	0.0290
Sex							
Male	8934/22,362 [40.0]	31	0.2840	<0.0001	0.76	0.67–0.85	<0.0001
Female	11,170/28,707 [38.9]	31	0.2840	<0.0001	1.32	1.17–1.49	<0.0001
Marital status							
Married	6888/20,496 [33.6]	17	0.1950	<0.0001	0.84	0.75–0.95	0.0040
Single	7173/18,764 [38.2]	17	0.1950	<0.0001	1.19	1.06–1.34	0.0040
Educational attainment							
<BSC	12,130/22,950 [52.9]	22	0.5260	<0.0001	1.30	1.03–1.65	0.0300
≥BSC	17,532/41,182 [42.6]	22	0.5260	<0.0001	0.77	0.61–0.97	0.0300
Religion							
Christian	1053/2124 [49.6]	5	<0.0001	0.4380	1.17	1.01–1.35	0.0340
Muslim	1265/3961 [31.9]	6	0.5110	<0.0001	1.39	0.85–2.26	0.1860
Hindu	16/129 [12.4]	2	1.5700	0.0710	0.28	0.02–3.40	0.3150
Employment							
Not working	1704/4455 [38.2]	10	0.1790	0.0090	1.20	1.02–1.42	0.0300
Working	5883/16,413 [35.8]	10	0.1790	0.0090	0.83	0.71–0.98	0.0300
Healthcare-related job	2886/8313 [34.7]	10	0.3340	<0.0001	0.68	0.52–0.89	0.0040
Socioeconomic status							
Low income	1320/2939 [44.9]	7	0.4840	<0.0001	1.31	0.88–1.94	0.1790
Middle income	1217/3220 [37.8]	7	1.2050	<0.0001	0.61	0.25–1.52	0.2900
High income	1427/2515 [56.7]	7	1.2860	<0.0001	1.28	0.49–3.38	0.6140
Urbanicity							
Urban	9192/28,583 [32.2]	15	0.4500	<0.0001	0.92	0.72–1.18	0.5070
Rural	3128/8338 [37.5]	15	0.4500	<0.0001	1.09	0.85–1.39	0.5070
Children at home	1207/4595 [26.3]	8	1.4040	<0.0001	0.37	0.14–0.99	0.0490
Aged people at home	456/1542 [29.6]	5	0.2760	0.0140	1.07	0.78–1.45	0.6920
Household number (n)							
≤2	930/3192 [29.1]	5	0.3900	<0.0001	0.94	0.64–1.36	0.7270
3–4	564/2067 [27.3]	5	0.2290	0.0110	0.89	0.69–1.14	0.3510
≥5	278/712 [39.0]	4	0.1680	0.1620	1.36	1.13–1.63	0.0010
Family members with medical backgrounds	464/1382 [33.6]	2	0.0410	0.3170	0.92	0.78–1.07	0.2770
Wearing masks all the time	1523/6606 [23.1]	6	0.5570	<0.0001	0.61	0.36–1.05	0.0720
Constantly washing hands	1209/4974 [24.3]	4	0.8900	<0.0001	0.45	0.18–1.16	0.0980
Keep physical distancing	213/745 [28.6]	3	0.8370	0.0010	0.23	0.08–0.65	0.0050
Smoker	665/2236 [29.7]	5	0.0590	0.3360	1.13	0.99–1.29	0.0610
History of chronic disease(s)	3828/8197 [46.7]	17	0.1840	<0.0001	0.91	0.80–1.03	0.1420
Ever tested for COVID-19	670/4430 [15.1]	6	0.4340	<0.0001	0.46	0.31–0.68	<0.0001
Personal history of COVID-19 diagnosis	4114/7733 [53.2]	15	0.6090	<0.0001	0.94	0.66–1.33	0.7150
Family member/friend ever diagnosed with COVID-19	1192/3759 [31.7]	7	0.2960	<0.0001	0.83	0.63–1.09	0.1730
Hospitalization due to COVID-19 among people in the same social circle	69/621 [11.1]	2	<0.0001	0.9770	0.57	0.37–0.88	0.0110
Death due to COVID-19 among people in the same social circle	63/537 [11.7]	3	<0.0001	0.9450	0.73	0.49–1.08	0.1160
COVID-19 vaccines are not safe	628/1595 [39.4]	6	0.7000	<0.0001	2.24	1.21–4.14	0.0100
Influenza vaccination in the past few years	3481/10,687 [32.6]	11	0.3460	<0.0001	0.46	0.36–0.58	<0.0001

OR: odds ratio; CI: confidence interval; NS: number of studies; *p* Het: *p* heterogeneity; BSC: Bachelor of Science.

## Data Availability

All data underlying the results are available at the Supplementary Files (https://doi.org/10.6084/m9.figshare.20055539.v3). Accessed on 11 June 2022.

## Supplementary Materials

The following supporting information can be downloaded at: https://www.mdpi.com/article/10.3390/vaccines10081356/s1, Supplementary S1: PRISMA 2020 Checklist.
